# Synthesis of thermoresponsive oligo(ethylene glycol) polymers through radical ring-opening polymerization of vinylcyclopropane monomers†

**DOI:** 10.1039/c9ra10721e

**Published:** 2020-01-13

**Authors:** Jovana Stanojkovic, Junki Oh, Anzar Khan, Mihaiela C. Stuparu

**Affiliations:** Division of Chemistry and Biological Chemistry, School of Physical and Mathematical Sciences, Nanyang Technological University 21-Nanyang Link 637371 Singapore mstuparu@ntu.edu.sg; Department of Chemical and Biological Engineering, Korea University Seoul 02841 South Korea anzar@korea.ac.kr; School of Materials Science and Engineering, Nanyang Technological University 50 Nanyang Avenue 639798 Singapore

## Abstract

Polyvinylcyclopropanes are an old class of polymers typically known for their low polymerization-induced shrinkage properties. In this work, we show that they are capable of exhibiting a thermally triggered aggregation process in aqueous solutions. The phase transition is sharp, tunable within the temperature range of 25–46 °C, and relatively insensitive to environmental conditions. It is anticipated that this preliminary study will shine new light on polyvinylcyclopropanes and lead to new avenues in their studies and future application.

## Abstract 2

Polyvinylcyclopropanes are designed to exhibit a thermally triggered aggregation process in aqueous solutions.

## Graphical abstract

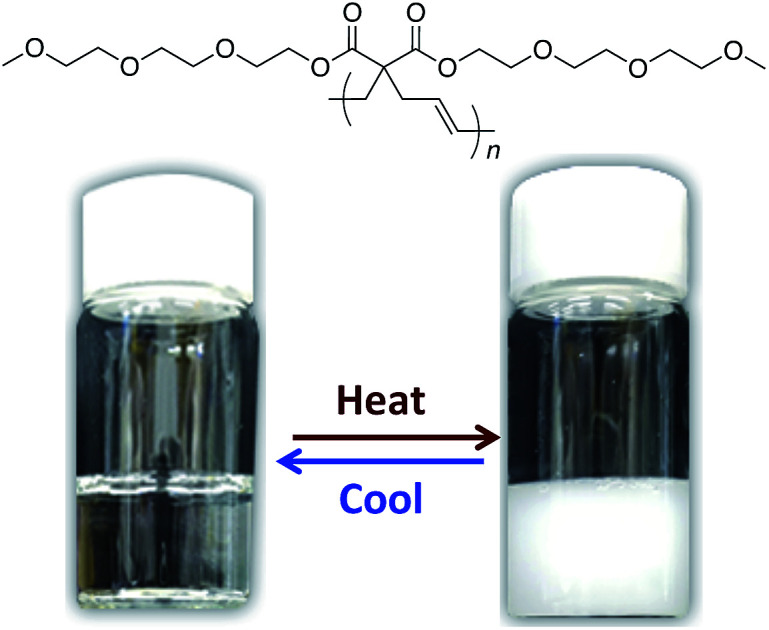

The 1,1-disubstituted vinylcyclopropane monomers can be polymerized by a free radical reaction mechanism wherein the vinyl group serves as a radical acceptor and the ring-strain provides the necessary thermodynamic driving force for propagation through a β-fragmentation pathway (Scheme 1).^1^ Such a ring-opening polymerization is of considerable value in the production of low-volume shrinkage materials. It is known that in non-cyclic monomers such as styrenes, acrylates, or methacrylates, the polymerization process decreases the van der Waals distance between the two monomers as they become connected to each other in the polymer chain through a shorter covalent bond. Therefore, the material contracts as a whole. In the case of cyclic monomers, however, the bond formation is accompanied by a bond cleavage and opening of the ring structure. This results in partial compensation of the volume contraction and reduction in the extent of material shrinkage.^2^ For this reason, the main research focus on vinylcyclopropanes is restricted to coatings, precision-molding, and cavity-filling (*e.g.*, dental implants) applications. This restriction is noteworthy as functionalized vinylcyclopropane monomers can be synthesized in 1–2 simple and high-yielding steps and polymerized to high conversions under relatively mild conditions. We envisaged that endowing stimuli responsive properties to this unique polymer family would enhance its scope and appeal in polymer and biomaterials sciences. To achieve this goal, we developed a very simple molecular design through which the polyvinylcyclopropane backbone becomes prone to aggregation in water as the solution temperature is raised. Inspired by Lutz's methacrylate polymers,^3^ in our design, two triethylene glycol units are attached to the vinylcyclopropane scaffold.^4^ These side-chains allow the polymer chain to be solvated in water through hydrogen bonding interactions at room temperature. However, as the solution temperature is raised, the favorable interactions between the water molecules and the polymer chains weaken and an entropically-driven dehydration process takes place. The net result of this process is the precipitation of the polymer chains out of the solution. Polymers with such sensitivity to temperature are known as thermoresponsive polymers.^5^ Due to this property, they find a number of bio-relevant applications.^6^

**Scheme 1:**
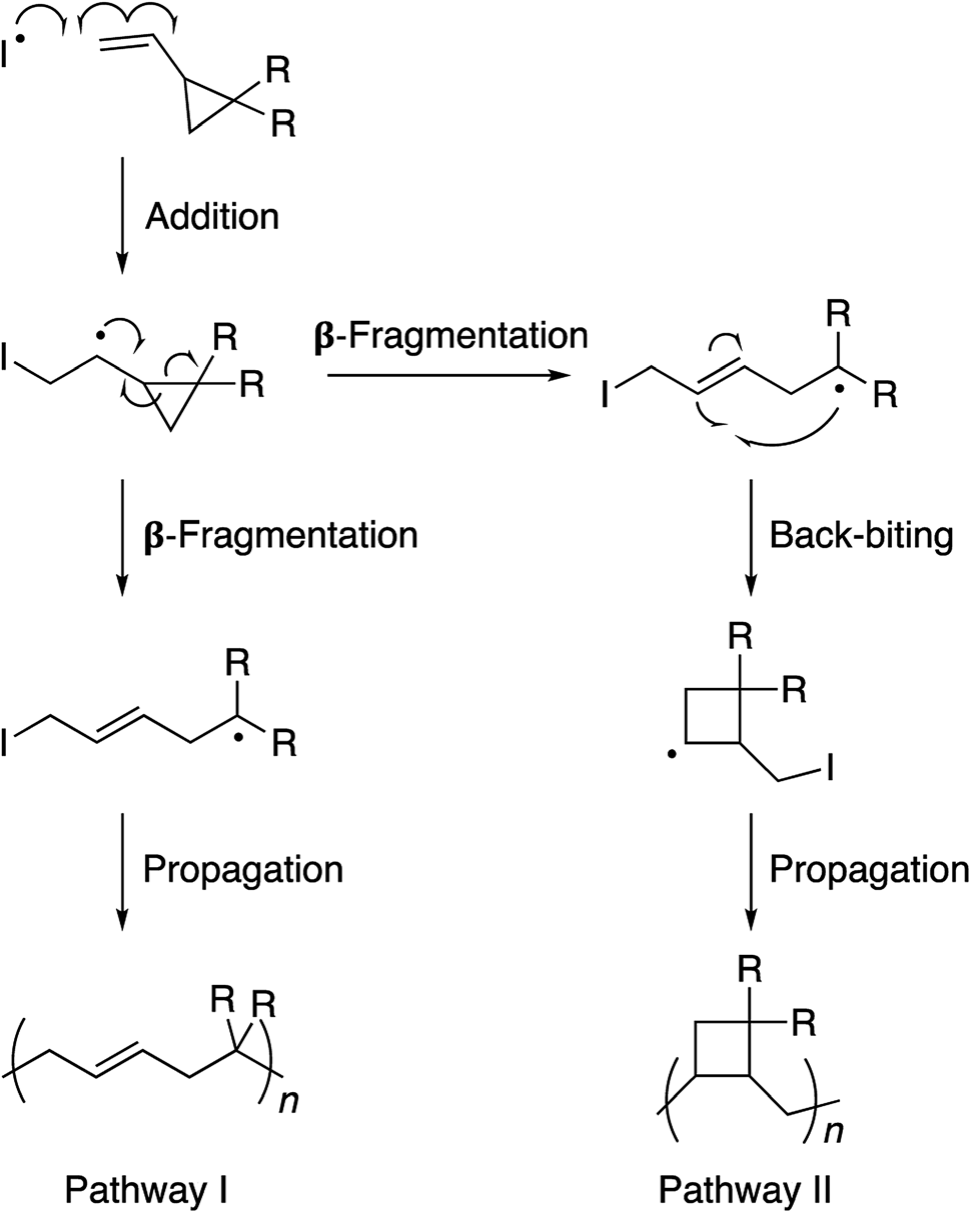
Polymerization of vinylcyclopropane monomers *via* free radical chemistry. Pathway I is preferred due to the propagating radical stabilization by the electron withdrawing R substituents.

In considering stimuli-responsive vinylcyclopropane polymers, to our knowledge, only two examples are known thus far.^7,8^ In 2003, Ritter and coworkers presented supramolecular complexation between cyclodextrin and a vinylcyclopropane monomer carrying a poly(*N*-isopropylacrylamide) (PNIPAM) side-chain.^7^ For steric reasons, the macromonomer could only dimerize and a copolymerization approach with NIPAM was necessary to obtain polymers of significant molecular weights. These polymers, due to the NIPAM-rich structure, were thermoresponsive in a temperature range of 28–32 °C. In the other study, Theato and coworkers presented a post-polymerization modification approach^9^ of activated-ester functionalized vinylcyclopropanes to prepare alkyl-amide side-chain polymers.^8^ The final polymers were not soluble in water, but a thermoresponsive character could be established in ethanol or aqueous ethanol solutions. Our molecular design is different from Ritter's approach such that the monomer is of considerably low molecular weight and the polymerization is not hindered by the steric bulk of the side-chains. It also guarantees a defect-free and perfect repeating unit structure, which is often difficult to achieve in a post-polymerization modification approach. Finally, unlike the aforementioned studies, we establish systematic tunability of the thermal transition in water by incorporation of a second monomer in the molecular design thus providing a general platform for the future development of a variety of thermoresponsive materials based on the vinylcyclopropane polymer family.

The synthesis of the targeted vinylcyclopropane monomer 1 was accomplished in two simple steps (Scheme 2). The first step involved esterification reaction between triethylene glycol monomethyl ether and malonyl chloride furnishing di-triethylene glycol monomethyl ether malonate in 92% yield (Fig. S1†). A base-catalyzed reaction between 1,4-dibromobutene and the malonate leads to the formation of monomer 1 in 80% yield. In ^1^H-NMR, proton resonances from the cyclopropane ring of monomer 1 could be observed at 2.5 and 1.5–1.7 ppm (Fig. S2†) while the signal from the olefin protons could be located in the region of 5–5.5 ppm (Fig. S2†).

**Scheme 2:**
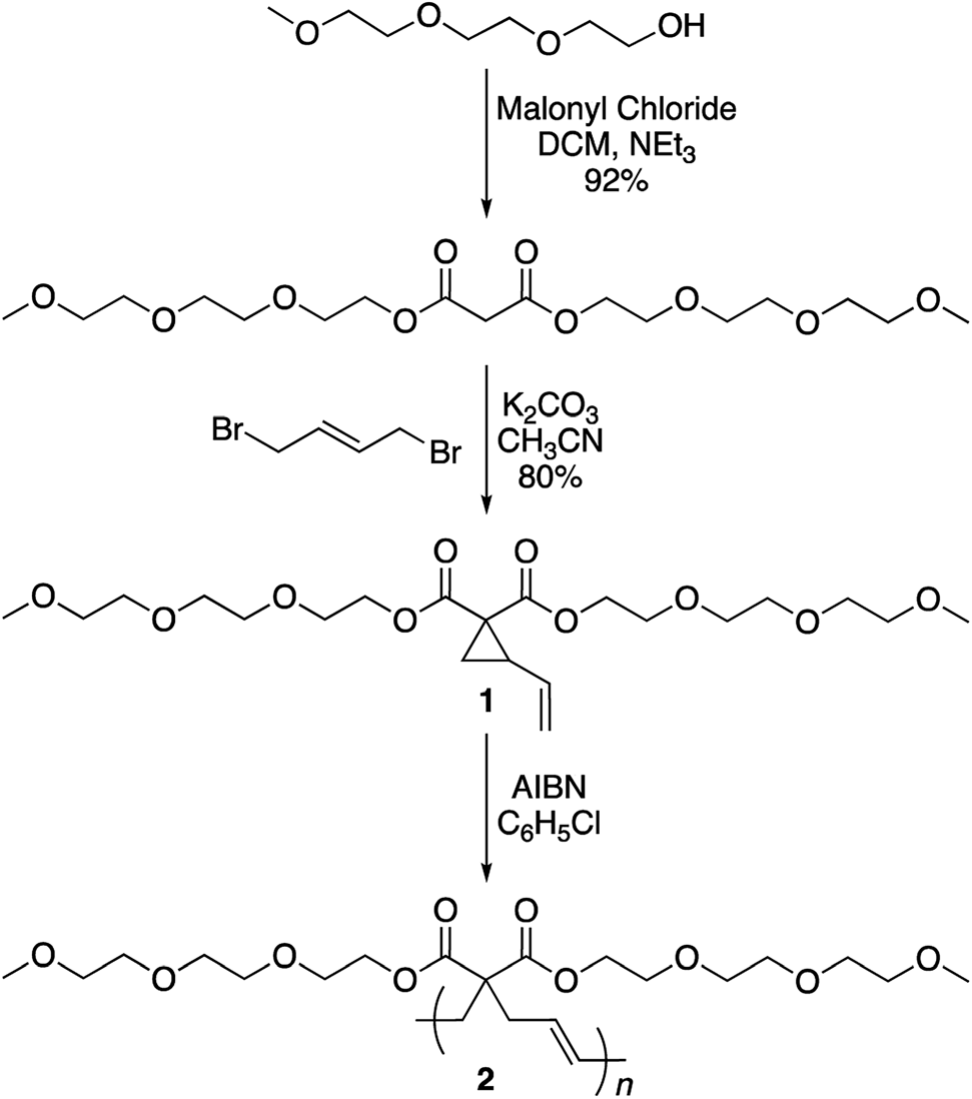
Synthesis of monomer 1 and polymer 2.

Monomer 1 could be polymerized using azobisisobutyronitrile (AIBN) as a free radical initiator in chlorobenzene. The polymerization proceeds well at a reaction temperature of 65 °C. It is known that such mild conditions favors 1,5-type radical addition.^2*c*^ Indeed, ^1^H-NMR confirmed that the polymerization proceeded through the 1,5-adduct as resonances from the cyclobutane ring, which would form by a competing radical back-biting mechanism (pathway II in Scheme 1), could not be seen in the area of 2–2.5 ppm (Fig. 1).^2*c*^ Gel permeation chromatography (GPC) analysis against polystyrene standard indicated the number average molecular weight to be 10 000 g mol^−1^ with a high dispersity index (*M*_w_/*M*_n_ = 2) as expected for a conventional free radical polymerization process (Fig. S3†).

**Fig. 1:**
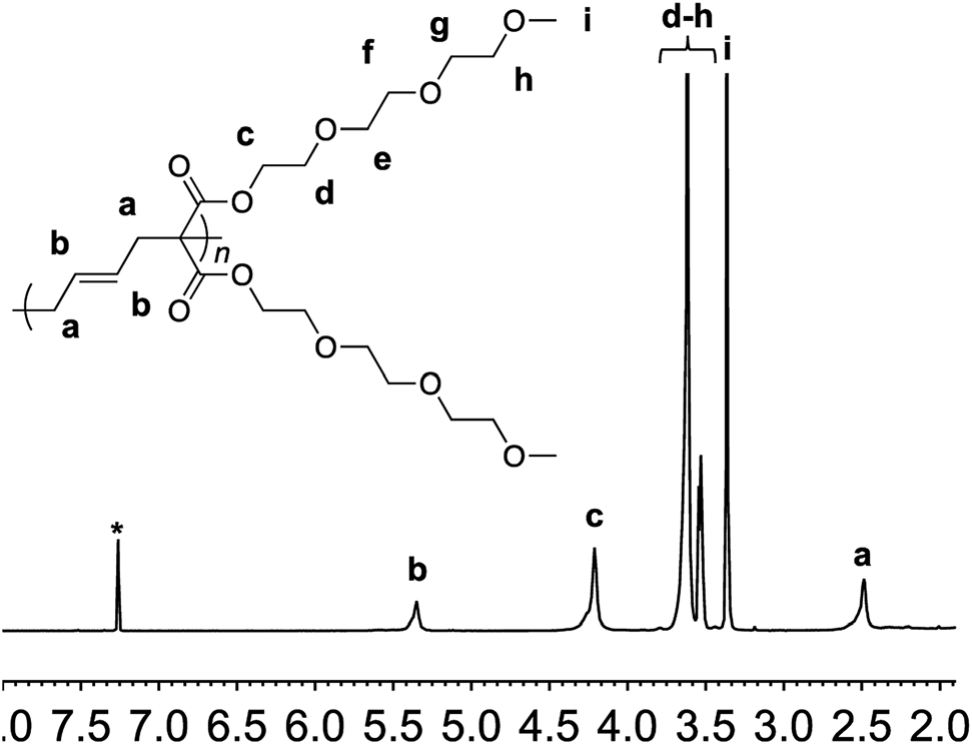
^1^H-NMR of polymer 2 in deuterated chloroform. Residual solvent signal is shown with an asterisk.

Having facile access to the polymer, a transmittance study was undertaken to evaluate its thermoresponsive character. This analysis indicated that the transition from the fully hydrated state to the aggregated state occurred at 46.5 °C (Fig. 2). In this study, the phase transition temperature is defined as the temperature of the inflection point of the heating curve.

**Fig. 2:**
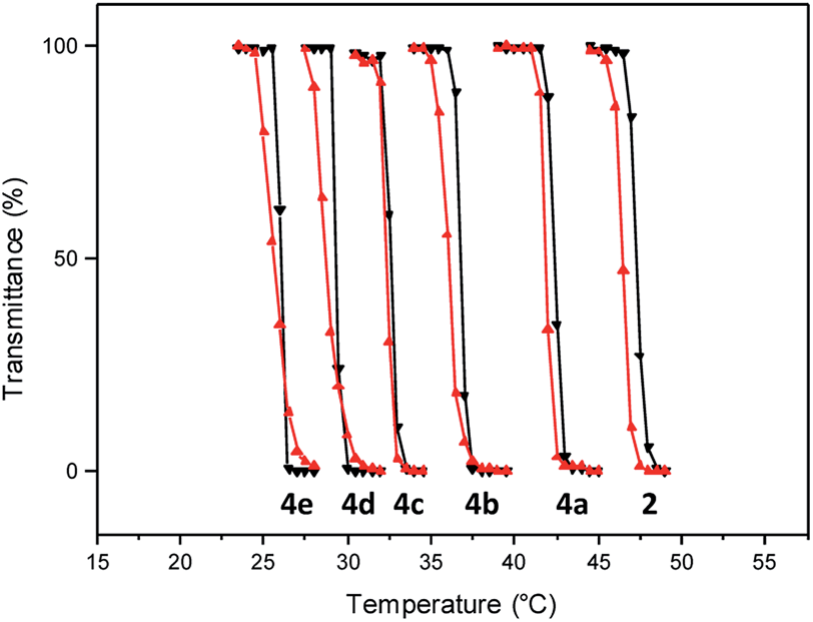
Transmittance at 600 nm as a function of temperature (0.5 °C min^−1^) for aqueous solution (10 mg mL^−1^) of polymers 2 and 4 (heating cycles, black triangles; cooling cycles, red triangles).

Variable temperature NMR provides an additional tool to examine the thermal collapse process of the polymer chains. At room temperature, for instance, the spectrum of polymer 2 shows clear and intense proton resonances from the side-chains as well as the backbone in deuterated water. This indicates that both segments of the polymer chain are well solvated and freely mobile (Fig. 3). However, as the solution temperature is raised, the proton resonances from the backbone shifts downfield, broadens, and finally merge with the baseline. The proton resonances from the side-chains exhibit a similar pattern. These changes indicate that at higher temperatures, the overall chain mobility decreases and the polymer chains start to aggregate.

**Fig. 3:**
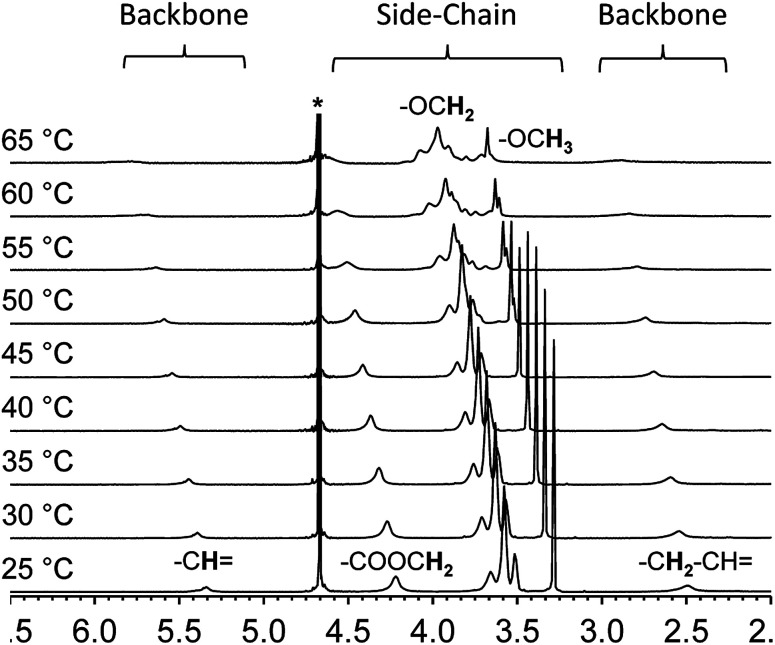
Variable temperature ^1^H-NMR of polymer 2 in deuterated water (10 mg mL^−1^).

Since the thermal phase transition depends upon interaction of water molecules with the polymer chain, it could be tuned through incorporation of hydrophobic segments into the polymer structure. It is anticipated that as the hydrophobic fraction increases, the hydrogen bonding ability of the polymer chain decreases. Therefore, attractive interactions between the polymer chain and water molecules can be weakened at a lower temperature range and the polymer chain aggregation can be accelerated.

To achieve this goal, hydrophobic monomer 3 carrying ethyl side-chains was synthesized in one step from commercially available diethyl malonate (Scheme 3 and Fig. S4†).^10^ A copolymerization between monomers 1 and 3 afforded polymer 4 with varying compositions. As can be seen in the ^1^H-NMR spectra, the copolymers displayed the ethyl group signals at 1.3 and 4.2 ppm (Fig. 4). Area integration analysis indicated that polymers 4a, 4b, 4c, 4d, and 4e were composed of 9, 17, 23, 29, and 33% hydrophobic monomer, respectively. The molecular weights of the copolymers ranged from 50 000 to 80 000 g mol^−1^ with dispersity index of 2.1–2.8.

**Scheme 3:**
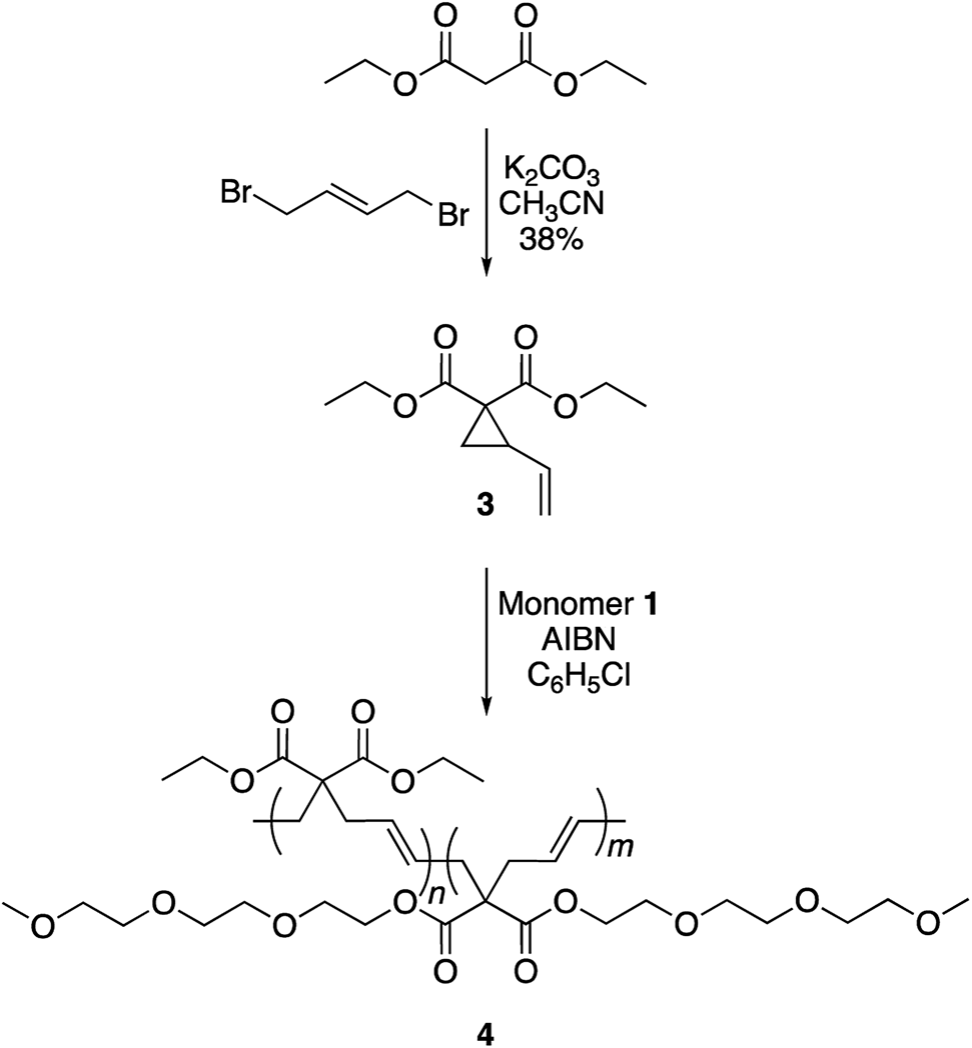
Synthesis of copolymer 4.

**Fig. 4:**
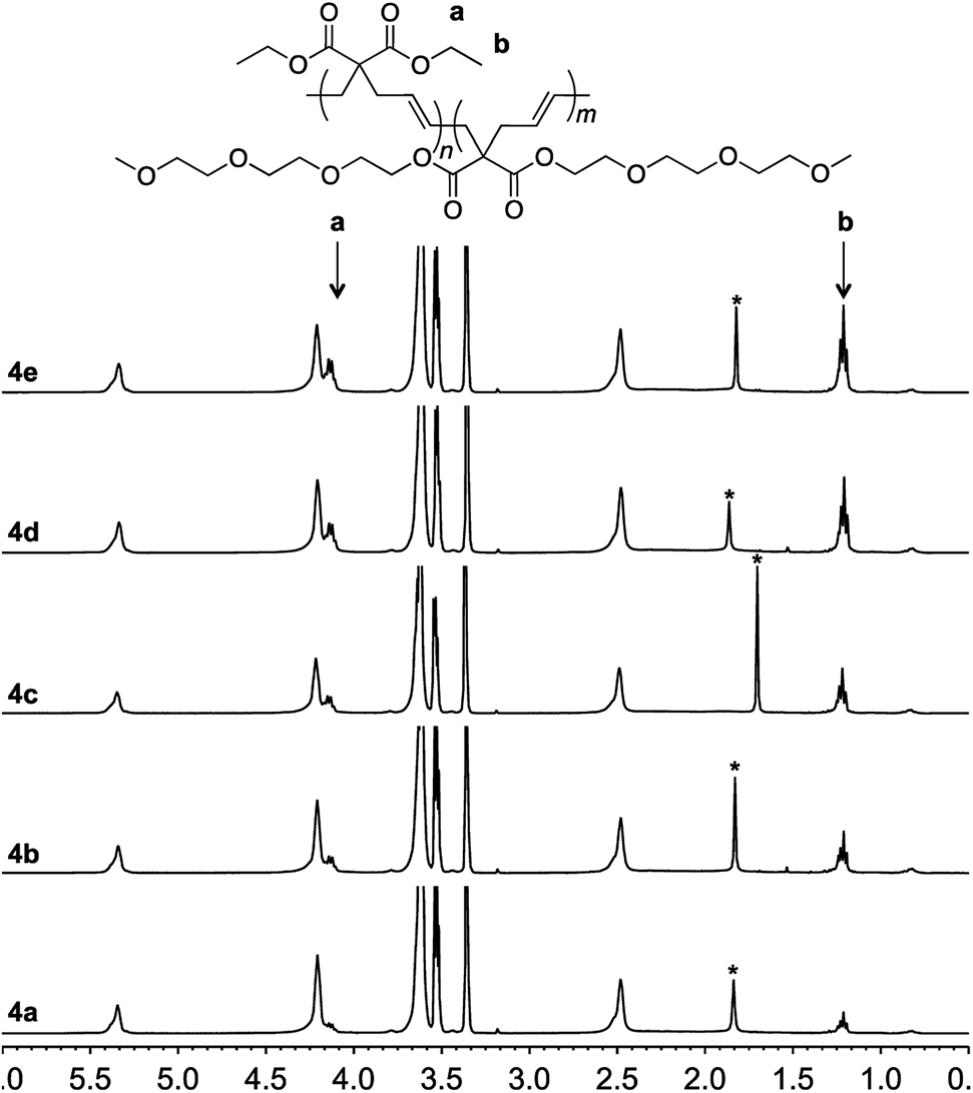
^1^H-NMR of polymer 4a–4e in deuterated chloroform. The signal from water is marked with an asterisk.

Incorporation of the hydrophobic repeating unit in the polymer structure had significant influence on the phase transition of the polymers. Polymers 4a with the least amount of the hydrophobic segment displayed a thermal transition at 41.8 °C (Fig. 2 and S5†). An increase in the content of the hydrophobic monomer led to a further decrease in the thermal phase transition in polymers 4b (36.0 °C), 4c (32.5 °C), 4d (29.0 °C), and 4e (25.5 °C).

From these copolymers, polymer 4b with a transition temperature of 36 °C was chosen to further examine its aggregation behavior through turbidimetry. Initially, a variable concentration study was undertaken. This study indicated that the transition temperature remained unchanged from 1 wt% material concentration to 0.8 and 0.6 wt% concentrations (Fig. S6†). Below this concentration, the transition temperature increases slightly with decreasing concentration. As higher concentrations are expected to facilitate aggregation, the results seemed logical. However, the change in the transition temperature is within 2 °C and can be considered negligible.^3*b*^

Finally, influence of a salt (sodium chloride) was evaluated on the thermal transition property of the polymer 4b (Fig. S7†). In general, the transition temperature decreases with an increase in the salt concentration. This is anticipated as the salt helps in the dehydration process. Once again, however, the inflection point temperature varied only slightly within a range of 2 °C which can be again considered negligible.^3*b*^

In summary, vinylcyclopropane monomers carrying triethylene glycol or ethyl side-chains can be prepared in 1–2 synthetic steps. A free radical polymerization affords polymers through cyclopropane ring-opening reaction. ^1^H-NMR indicates that the polymers are defect-free and form exclusively through 1,5-radical addition pathway. The synthesized polymers are prone to aggregation as temperature of the aqueous solution is raised. The phase transition temperature can be adjusted through changing molecular composition of the copolymer. In general, an increase in the fraction of the hydrophobic ethyl side-chain-carrying monomer reduces the transition temperature. The thermal response is found to be relatively insensitive to the environmental conditions such as a change in material concentration or presence of salt in the aqueous solution. It is anticipated that these preliminary results will invoke further investigations into thermoresponsive properties of vinylcyclopropanes, thus far appreciated mostly for their low shrinkage properties, and lead to new applications for this old polymer class.

## Conflicts of interest

There are no conflicts to declare.

## Supplementary Material

RA-010-C9RA10721E-s001
