# Supplementary material for: Synthesis of thermoresponsive oligo(ethylene glycol) polymers through radical ring-opening polymerization of vinylcyclopropane monomers
Source: RSC Adv. 2020 Jan 13;10(4):2359–63. doi: 10.1039/c9ra10721e (PMC9048585; doi:10.1039/c9ra10721e)
Supplement: RA-010-C9RA10721E-s001 [file RA-010-C9RA10721E-s001.pdf]

Electronic Supplementary Material (ESI) for RSC Advances.
This journal is © The Royal Society of Chemistry 2020

Synthesis of Thermoresponsive Oligo(ethylene glycol) Polymers Through Radical Ring Opening Polymerization of Vinylcyclopropane Monomers

Jovana Stanojkovic, Junki Oh, Anzar Khan and Mihaiela C. Stuparu

Emails: anzar@korea.ac.kr and mstuparu@ntu.edu.sg

Experimental Details

Synthesis: All commercial reagents were used as received from commercial suppliers without further purification. All reactions relating to air- and moisture-sensitive reagents were performed in vacuum-dried reaction vessels under a nitrogen atmosphere using dried solvents. Column chromatography was carried out on silica gel 40–63 mesh as the stationary phase. Reactions were monitored by thin layer chromatography (TLC) with silica gel coated aluminum plates (60 F254, Merck) and visualized by $KMNO_4$ stain. ^{1}H NMR spectra were recorded at 396 or 400 MHz JEOL ECA instruments (and the corresponding frequencies for ^{13}C) in $CDCl_3$ unless otherwise noted. Chemical shifts are given in ppm ($CDCl_3$ ^{1}H: 7.26 ppm, ^{13}C: 77.23 ppm). ^{1}H-NMR splitting patterns are designated as s (singlet), d (doublet), t (triplet), q (quartet), dd (doublet of doublets), and m (multiplet). Gel permeation chromatography (GPC) was carried out in THF using a Waters system equipped with a refractive index detector and polystyrene standards.

Bis(triethylene glycol monomethyl ether) malonate: In a solution of triethylene glycol monomethyl ether (14 g, 0.085 mol, 3.0 equiv.) in dry DCM (40 mL), Et_3N (6.22 g, 0.061 mol, 2.2 equiv.) was added under N_2 atmosphere, followed by addition of malonyl chloride (4 g, 0.028 mol, 1.0 equiv). The reaction mixture was stirred at room temperature for 1 h and then quenched with H_2O, extracted with DCM, dried over Na_2SO_4, and concentrated under reduced pressure. The crude product was purified by SiO_2 column chromatography (EtOAc/MeOH = 100/3) to afford the product (10.2 g) in 92% yield. ^{1}H-NMR (500 MHz, $CDCl_3$) δ 4.20-4.18 (m, 4H), 3.62-3.60 (m, 4H), 3.57-3.50 (m, 12H), 3.46-3.44 (m, 4H), 3.34 (s, 2H), 3.27 (s, 6H).

1,1-Bis(triethylene glycol monomethyl etheroxycarbonyl)-2-vinylcyclopropane (**1**): To a stirring solution of bis(triethylene glycol monomethyl ether) malonate (4 g, 0.01 mol, 1 equiv.), K_2CO_3 (4.19 g, 0.03 mol, 3 equiv.) and 18-crown-6 (792 mg, 0.003 mol, 0.3 equiv.) in CH_3CN (75 mL), a solution of 1,4-dibromo-2-butene (2.6 g, 0.012 mol, 1.2 equiv.) in CH_3CN (10 mL) was added drop wise and the reaction mixture was stirred for 21 h at room temperature. Afterwards, the reaction was quenched with 0.5 M HCl (50 mL), extracted with DCM, dried over Na_2SO_4, and concentrated under reduced pressure. The obtained crude product was purified by SiO_2 chromatography (EtOAc) to give **1** (3.65 g) in 80% yield. ^{1}H-NMR (500 MHz, $CDCl_3$) δ 5.46-5.37 (m, 1H), 5.26-5.21 (m, 1H), 5.09-5.07 (m, 1H), 4.24-4.21 (m, 4H), 3.65-3.57 (m, 16H), 3.50-3.47 (m, 4H), 3.32 (s, 6H), 2.58-4.52 (m, 1H), 1.69-1.66 (m, 1H), 1.56-1.52 (m, 1H). ^{13}C NMR (100 MHz, $CDCl_3$) δ 169.31, 167.06, 133.02, 118.60, 71.93, 70.60, 70.59, 70.54, 68.90, 68.81, 64.61, 64.41, 58.95, 35.78, 31.49, 20.57.

Polymer **2**: In a pre-dried glass tube containing dried monomer **1** (100 mg, 0.222 mmol, 1 equiv.) was added a solution of AIBN in degassed chlorobenzene (100 µl of 4.4×10^{-4} mol/L solution, 0.0446 mmol, 20 mol%). The polymerization mixture was stirred overnight at 65 °C. Afterwards, the polymerization tube was opened and the

polymerization mixture was precipitated and washed with Et_2O to give polymer **2** in quantitative yields. ^{1}H-NMR (500 MHz, $CDCl_3$) δ 5.34 (broad s, 2H), 4.24-4.16 (broad s, 4H), 3.73-3.55 (m, 16H), 3.53-3.51 (m, 4H), 3.35 (s, 6H), 2.47 (broad s, 4H). ^{13}C NMR (100 MHz, $CDCl_3$) δ 170.17, 128.59, 71.97, 70.62, 70.54, 70.47, 68.83, 64.35, 58.97, 58.93, 35.85. GPC(THF): M_n = 10000, PDI (M_w/M_n) = 2.

1,1-Bis(ethoxycabonyl)-2-vinylcyclopropane **3**: To a THF (12.5 mL) solution of 1,4-dibromo-2-butene (3.21 g, 15 mmol, 1 equiv.) was added diethyl malonate (2.40 g, 15 mmol, 1 equiv.) under N_2. The mixture was then cooled to 0 ^{o}C and a solution of NaH (0.72 g, 30 mmol, 2 equiv.) in THF (10 mL) added. The resulting reaction mixture was stirred under reflux (78 ^{o}C) for 18 h. After cooling, the reaction mixture was quenched with H_2O and extracted with DCM. The combined organic layer was dried over Na_2SO_4 and concentrated under reduced pressure. The obtained crude mixture was purified using SiO_2 column chromatography (DCM/hexane = 9/1) to give product **3** (1.2 g) in 38% yield. ^{1}H-NMR (300 MHz, $CDCl_3$) δ 5.46-5.39 (m, 1H), 5.30-5.27 (m, 1H), 5.14-5.11 (m, 1H), 4.26-4.12 (m, 4H), 2.60-2.54 (m, 1H), 1.69-1.67 (m, 1H), 1.56-1.53 (m, 1H), 1.26 (t, J = 7.1 Hz, 6H). ^{13}C NMR (100 MHz, $CDCl_3$) δ 169.62, 167.36, 133.16, 118.36, 61.56, 61.39, 35.92, 31.02, 20.28, 14.16, 14.03.

Polymer **4**: In five separate polymerization tubes containing dried monomers **1** and **3** in different molar ratios was added a solution of AIBN in degassed chlorobenzene (20 mol%). The mixture was stirred overnight at 65 °C. The obtained polymers were precipitated and washed with Et_2O. The polymer composition was determined with the help of area integration analysis in ^{1}H-NMR spectroscopy. The molar feed ratios (**1**:**3**) of 10:1, 10:2, 10:3, 10:4, and 10:5 resulted into incorporation of monomer **3** in polymer **4** in approximately 9, 17, 23, 29, and 33%, respectively. ^{1}H-NMR (500 MHz, $CDCl_3$) δ 5.34 (broad s, 2H), 4.24-4.16 (broad s, 4H), 4.16 (q, 2H), 3.73-3.55 (m, 16H), 3.53-3.51 (m, 4H), 3.35 (s, 6H), 2.47 (broad s, 4H), 1.25 (t, 3H). ^{13}C NMR (100 MHz, $CDCl_3$) δ 170.21, 128.59, 71.97, 70.62, 70.55, 68.83, 64.34, 61.13, 58.96, 35.78, 14.13. **4a** GPC(THF): M_n = 71000, PDI (M_w/M_n) = 2.76, **4b** GPC(THF): M_n = 61000, PDI (M_w/M_n) = 2.83, **4c** GPC(THF): M_n = 51000, PDI (M_w/M_n) = 2.46, **4d** GPC(THF): M_n = 80000, PDI (M_w/M_n) = 2.1, **4e** GPC(THF): M_n = 64000, PDI (M_w/M_n) = 2.62.

Study of thermoresponsive behavior: Lambda 265 UV/Vis-spectrometer from Perkin-Elmer was used for transmittance measurements at 600 nm by heating the filtered (0.2 μm pore size filter) aqueous solution by 0.5 °C with 10 mg/mL polymer concentration. The sample temperature was measured with temperature probe (thermometer equipped with Probe k-type thermocouple (-160/+700°C, Φ1.0×300mm, AS1.5522.04 from AS ONE) in cuvette at each interval. The thermal equilibration time was set at 10 minutes. In this work, the thermal phase transition is given by the temperature of the inflection point of the heating curve.

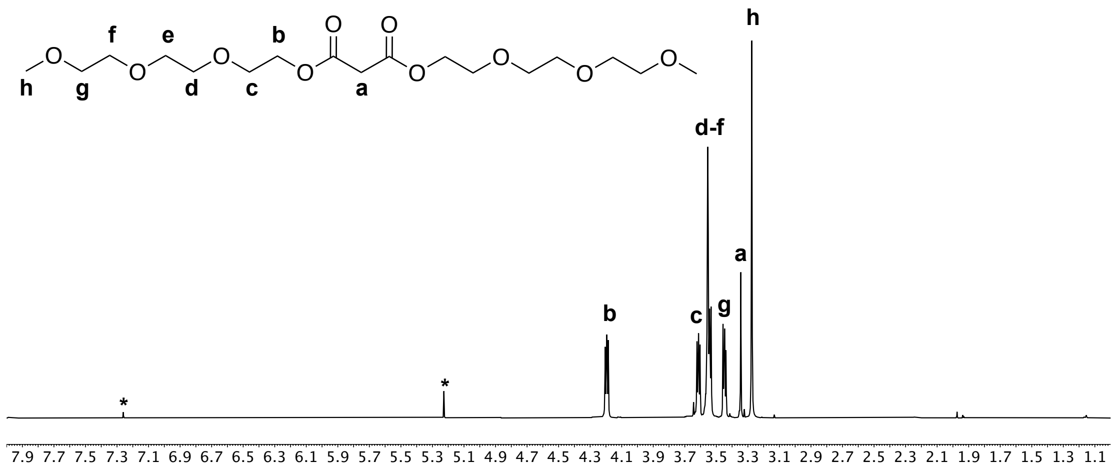

Figure S1. ^{1}H-NMR of bis(triethylene glycol monomethyl ether) malonate in deuterated chloroform. Residual solvent signals (dichloromethane and chloroform) are shown with an asterisk.

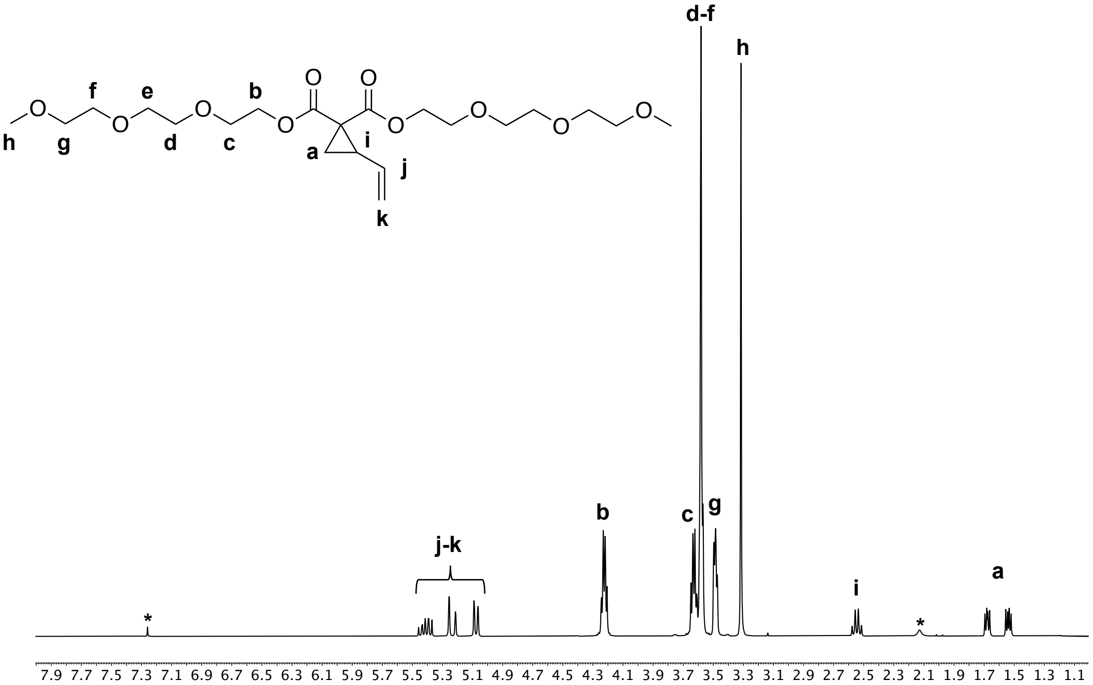

Figure S2. ^{1}H-NMR of monomer **1** in deuterated chloroform. Residual solvent signals (water and chloroform) are shown with an asterisk.

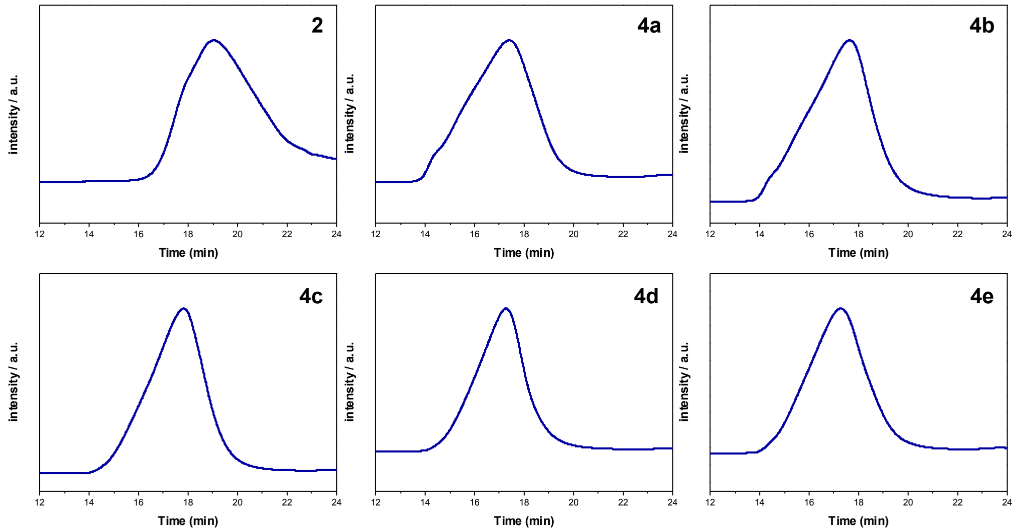

Figure S3. GPC traces for polymers **2** and **4** in THF against polystyrene standards.

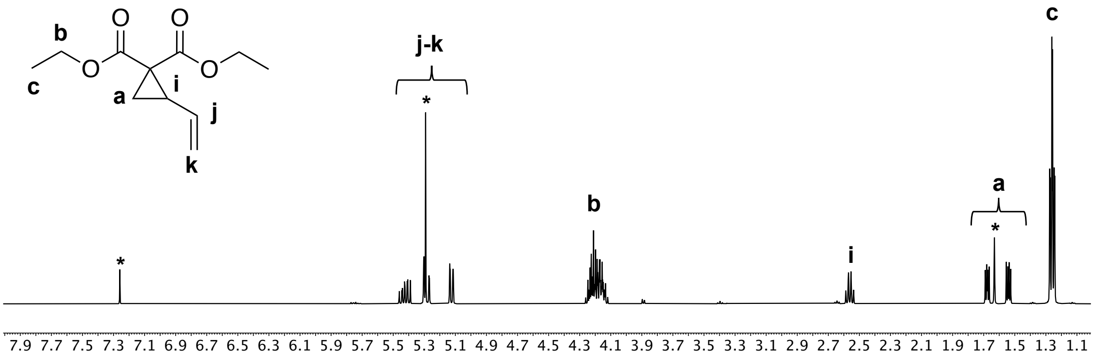

Figure S4. ^{1}H-NMR of monomer **3** in deuterated chloroform. Residual solvent signals (water, dichloromethane, and chloroform) are shown with an asterisk.

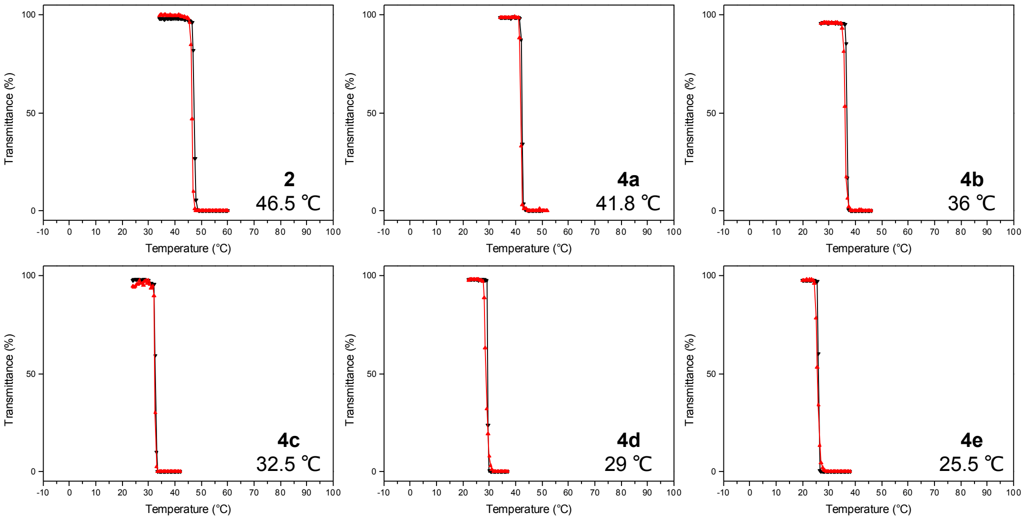

Figure S5. Transmittance at 600 nm as a function of temperature (0.5 °C/min) for aqueous solution (10 mg/mL) of polymers **2** and **4** (heating cycles, black triangles; cooling cycles, red triangles).

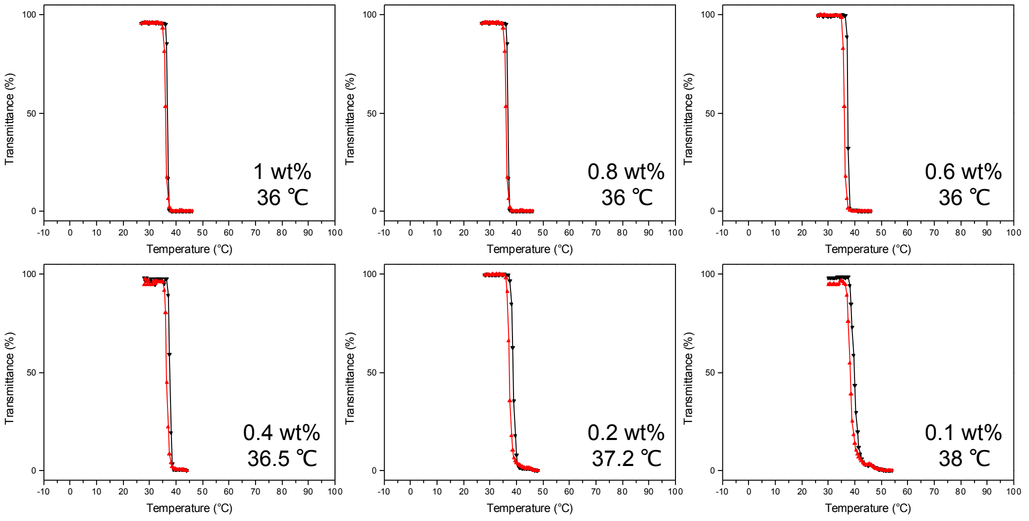

Figure S6. Transmittance at 600 nm as a function of temperature (0.5 °C/min) for aqueous solution of polymer **4b** (heating cycles, black triangles; cooling cycles, red triangles).

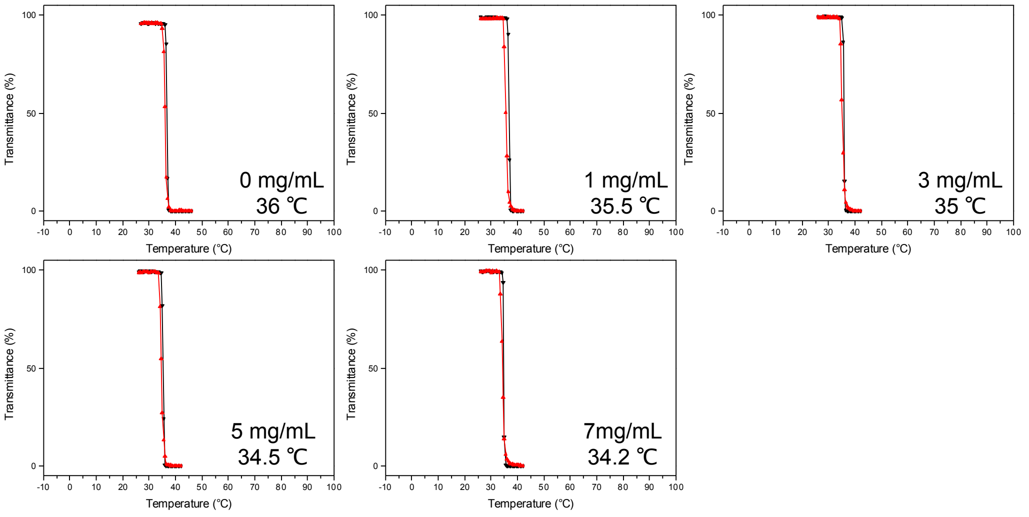

Figure S7. Transmittance at 600 nm as a function of temperature (0.5 °C/min) for aqueous solution (10 mg/mL) of polymers **4b** with NaCl (heating cycles, black triangles; cooling cycles, red triangles).
